# A Multifunctional Nanozyme Hydrogel with Antibacterial, Antioxidative, and Photo-Induced Nitric Oxide-Supplying Properties for Promoting Infected Wound Healing

**DOI:** 10.3390/pharmaceutics16121624

**Published:** 2024-12-22

**Authors:** Wen Zuo, Kunpeng Wei, Xinyi Zhang, Dongjing Wang, Haoyang Gong, Yanzhuo Zhang, Hui Wang

**Affiliations:** 1 Department of Pharmacy, Xuzhou Hospital of Traditional Chinese Medicine, Xuzhou 221003, China; zuowen201309@163.com; 2 Jiangsu Key Laboratory of New Drug Research and Clinical Pharmacy, Xuzhou Medical University, Xuzhou 221004, China; weikunpeng0407@163.com (K.W.); 17826069683@163.com (X.Z.); w15852019074@163.com (D.W.); 302111121024@stu.xzhmu.edu.cn (H.G.); yanzhuozhang@yeah.net (Y.Z.)

**Keywords:** hydrogel, multifunctional nanozyme, versatile delivery system, antibacterial, phototherapy

## Abstract

**Objectives:** To design a multifunctional nanozyme hydrogel with antibacterial, photo-responsive nitric oxide-releasing, and antioxidative properties for promoting the healing of infected wounds. **Methods:** We first developed ultra-small silver nanoparticles (NPs)-decorated sodium nitroprusside-doped Prussian blue (SNPB) NPs, referred to as SNPB@Ag NPs, which served as a multifunctional nanozyme. Subsequently, this nanozyme, together with geniposide (GE), was incorporated into a thermo-sensitive hydrogel, formulated from Poloxamer 407 and carboxymethyl chitosan, creating a novel antibacterial wound dressing designated as GE/SNPB@Ag hydrogel. The physical properties of a GE/SNPB@Ag hydrogel were systematically investigated. **Results:** After embedding the nanozyme and GE, the resulting GE/SNPB@Ag hydrogel retains its thermosensitive properties and exhibits sustained release characteristics. In addition to its catalase-like activity, the nanozyme demonstrates high photothermal conversion efficiency, photo-induced nitric oxide release, and antibacterial activity. In addition, the hydrogel exhibits favorable antioxidant properties and high biocompatibility. The results of animal experiments demonstrate that the composite hydrogel combined with laser irradiation is an effective method for promoting infected wound healing. **Conclusions:** In vitro and in vivo studies indicate that the resulting GE/SNPB@Ag hydrogel holds significant potential for the treatment of infected wounds and for further clinical applications.

## 1. Introduction

The skin serves as a vital protective barrier against external microorganisms. Infectious wounds not only cause local discomfort, but can also lead to complex health issues, resulting in systemic infections and potentially sepsis. As a result, infectious wounds have become a significant public health concern, adversely affecting the health and quality of life of many patients [1,2]. The continuous rise of multidrug-resistant (MDR) bacteria has increased the complexity of treating infectious wounds, posing substantial challenges to conventional treatment approaches [3]. The effective control strategies for managing acute wound infections include several key aspects: local wound management, infection control, the selection of appropriate dressings, and the maintenance of a moist healing environment [4]. Consequently, designing new antimicrobial dressings, innovative therapeutic methods, and personalized treatment strategies to enhance wound healing has become an urgent priority [5].

Hydrogels can provide a moist environment conducive to cell growth and metabolism. Their internal 3D porous structure not only allows for oxygen permeation and inhibits the proliferation of anaerobic bacteria, but also helps to increase the drug loading capacity and even promotes cell proliferation and migration [6,7]. As a result, hydrogel wound dressings have garnered widespread attention. Poloxamer 407 is extensively utilized in formulating in situ injectable thermos-responsive hydrogels. These hydrogels can prolong drug release, thereby reducing the necessary dosage and minimizing potential side effects. Moreover, Poloxamer 407-based hydrogels can be combined with various biocompatible and biodegradable polymers, such as alginate, polyvinylpyrrolidone, hyaluronic acid, carboxymethyl chitosan, and hydroxypropyl methylcellulose, to tailor their properties. For example, carboxymethyl chitosan can improve the bio-adhesive properties and moisture retention of hydrogels, rendering them beneficial for wound dressing applications [8,9]. However, traditional hydrogels are insufficient to meet the diverse needs of infected wound healing [10]. Therefore, there remains a pressing need to develop multifunctional hydrogels as wound dressings to prevent MDR bacterial infections, promote better wound healing outcomes, and minimize complications.

Photothermal therapy (PTT) is recognized as a safe and effective antibacterial strategy. Photothermal nanomaterials, such as polydopamine (PDA), copper sulfide, silver, black phosphorus, and palladium, convert light energy into heat upon exposure to near-infrared (NIR) irradiation, producing localized high temperatures [11,12,13,14,15]. This thermal increase can disrupt bacterial biofilms, ultimately leading to bacterial lysis and death. Moreover, PTT offers significant advantages, including non-invasiveness and spatiotemporal selectivity, and importantly, it is not constrained by drug resistance [16]. Therefore, hydrogels incorporating various photothermal components have already been utilized in the research of wound dressings.

Among photothermal nanomaterials, Prussian blue NPs exhibit both photothermal properties and peroxidase-like activity, enabling the effective catalysis of hydrogen peroxide into oxygen and water [17,18]. Sodium nitroprusside is a widely used medication that releases nitric oxide (NO). NO not only dilates blood vessels and increases local blood flow, but also promotes angiogenesis and enhances the proliferation and migration of fibroblasts and keratinocytes, playing an important role during wound healing [19]. Recent studies have demonstrated that sodium nitroprusside-doped Prussian blue (SNPB) nanoparticles (NPs) can harness the photothermal effect under NIR light irradiation to enhance the release of NO from sodium nitroprusside [20,21]. It is well known that silver NPs exhibit strong antibacterial activity. As an antibacterial agent, silver can disrupt the cell membrane, interfere with bacterial metabolism, and inhibit biofilm formation. Due to the diverse and complex antibacterial mechanisms of silver, its antimicrobial effects are less likely to lead to resistance. This characteristic facilitates its widespread application in wound dressings, medical device coatings, and preservatives [22,23,24]. Given the substantial advantages of SNPB NPs and silver NPs in antibacterial activity and wound healing, we designed a multifunctional nanozyme: ultra-small silver NPs-decorated SNPB NPs (SNPB@Ag). This innovative design seeks to integrate enhanced photothermal properties, specifically from SNPB and Ag, with photothermal-induced NO release and catalase activity into a single nanozyme.

Excessive reactive oxygen species (ROS) can prolong the inflammatory response, inhibit normal angiogenesis, impair tissue regeneration, and contribute to complications such as chronic non-healing wounds. The application of topical or systemic antioxidants effectively neutralizes excess ROS, reduces oxidative stress, and fosters a balanced healing environment [25]. Geniposide (GE), a cycloalkane ether glycoside predominantly found in gardenia, exhibits potent antioxidant properties that enable it to effectively eliminate excessive free radicals, thereby decreasing oxidative stress in cells [26]. Furthermore, GE can inhibit the production of inflammatory mediators, alleviating inflammatory responses [27]. Thus, combining nanozyme with GE may effectively mitigate the shortcomings of monotherapy and enhance treatment efficacy for infectious wounds. This approach is also distinct from other advanced antibacterial dressings, particularly those that utilize ROS generated by nanozymes to eliminate bacteria. Remarkably, to the best of our knowledge, the application of this multifunctional nanozyme and GE for the healing of infectious wounds has not been previously reported.

**Scheme 1:**
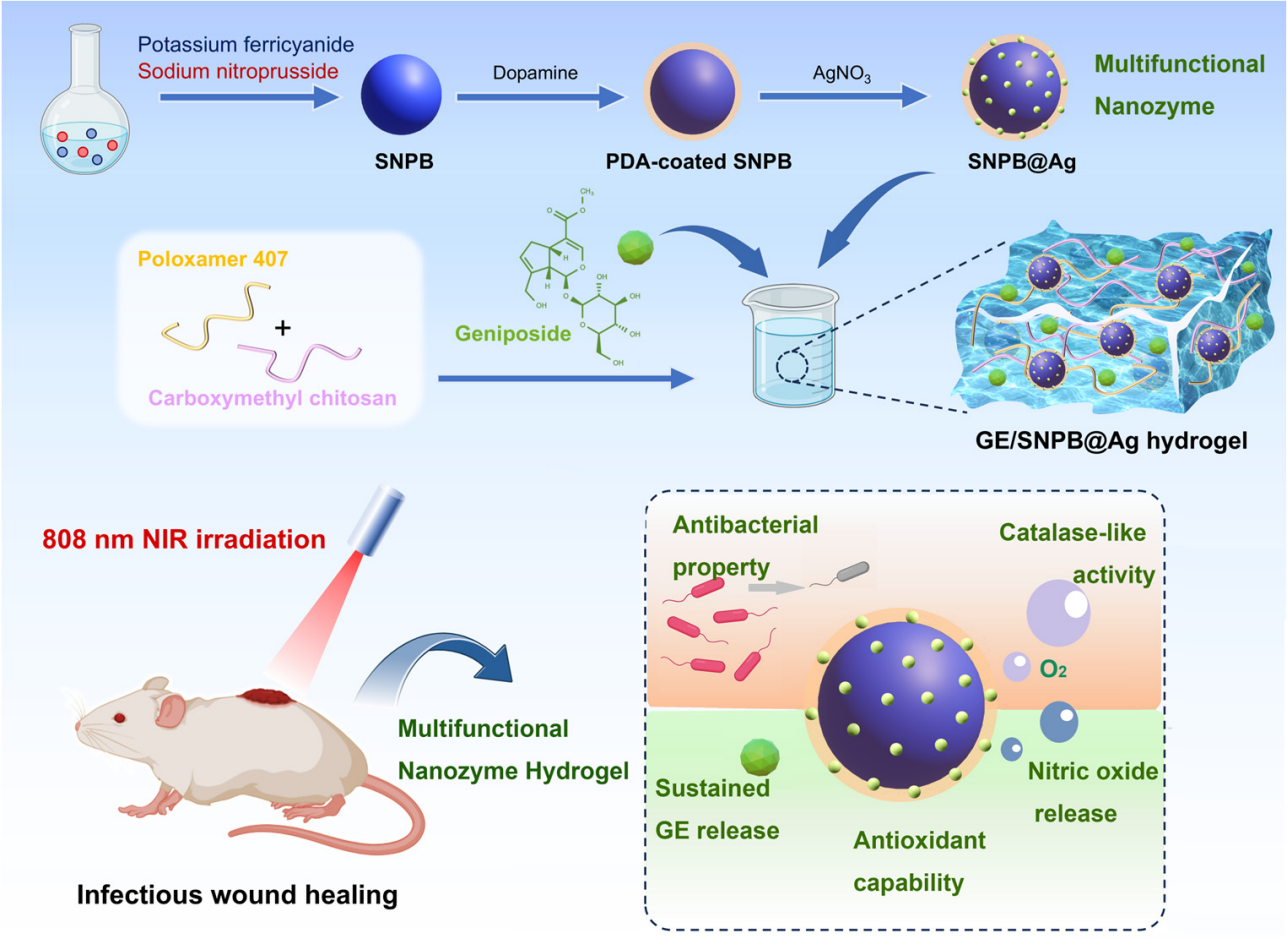
The schematic illustrates a two-step procedure for the preparation of the multifunctional nanozyme hydrogel and the mechanism through which this hydrogel promotes the healing of infectious wounds. SNPB, sodium nitroprusside-doped Prussian blue; SNPB@Ag, ultra-small silver nanoparticles-decorated SNPB; PDA, polydopamine; GE, geniposide; GE/SNPB@Ag hydrogel, geniposide and SNPB@Ag co-loaded hydrogel.

In this study, a novel multifunctional nanozyme (SNPB@Ag) was integrated with GE into a Poloxamer 407 and carboxymethyl chitosan-based thermo-sensitive hydrogel. Unlike the basic application of nanozymes or their straightforward incorporation into hydrogels, the developed multifunctional nanozyme hydrogel integrates the physical properties of injectable thermos-responsive hydrogels with photothermal technology, controlled modulation of nitric oxide and oxygen, and the elimination of excess ROS, resulting in a cohesive and multifunctional platform. This multifunctional nanozyme hydrogel demonstrates antibacterial, antioxidative, and photothermal-induced NO releasing properties, which are intended to promote infectious wound healing (Scheme 1). The multifunctional nanozyme hydrogel was evaluated on several characteristics, including its temperature responsiveness, rheological properties, sustained release capabilities, NIR-induced photothermal effects, nitric oxide and oxygen supply, and antibacterial and antioxidant activities, as well as its hemocompatibility and cytotoxicity. Furthermore, a mouse model of acute wound infections was utilized to investigate the therapeutic efficacy and application potential of the multifunctional nanozyme hydrogel.

## 2. Materials and Methods

### 2.1. Materials

Poloxamer 407 was supplied by BASF (Shanghai, China). Sodium nitroprusside, dopamine hydrochloride, potassium ferricyanide, polyvinylpyrrolidone (K30, PVP-K30), carboxymethyl chitosan (240 kDa, degree of deacetylation ≥90%, and substitution degree of 90%), and 2,2′-azino-bis (3-ethylbenzthiazoline-6-sulfonic acid) (ABTS) were obtained from Aladdin (Shanghai, China). Dulbecco’s modified Eagle’s medium (DMEM) and the Luria-Bertani (LB) were supplied by KeyGen (Jiangsu, Nanjing, China). GE (98%) was obtained from Macklin (Shanghai, China). Fetal bovine serum (FBS) was obtained from Gibco (Grand Island, NY, USA). The cell counting kit-8 (CCK-8) assay kits and Griess reagent kits were purchased from Beyotime (Shanghai, China). HaCaT cells were obtained from the Institute of Biochemistry & Cell Biology, Chinese Academy of Sciences (Shanghai, China). All the other reagents were of analytical grade.

### 2.2. Synthesis of SNPB@Ag NPs

The synthesis of SNPB@Ag NPs was accomplished in three steps. First, SNPB NPs were prepared following a previously reported method [20]. Specifically, 3 g of PVP-K30 was dissolved in 40 mL of 0.1 M hydrochloric acid (HCl) solution. After the addition of sodium nitroprusside (489 mg) and potassium ferricyanide (60 mg), the mixture was stirred continuously at 80 °C for 12 h. After this stirring period, the resulting blue precipitates were rinsed with distilled water and ethanol to eliminate excess PVP coating. Second, a thin coating of PDA was formed on the synthesized SNPB NPs. Typically, 20 mg of Tris-HCl and 9 mg of dopamine hydrochloride were dissolved in 60 mL of distilled water. Next, the previously obtained SNPB NPs were quickly added to this mixed solution, followed by ultrasonication for 10 min and continuous stirring for 2 h. The formed PDA-coated SNPB NPs were centrifuged (12,000× *g*, 15 min) and then rinsed with distilled water. Third, the ultra-small Ag NPs were deposited onto the PDA-coated SNPB NPs to synthesize the composite material, denoted as SNPB@Ag NPs. Briefly, the PDA-coated SNPB NPs (30 mg) were redispersed in 25 mL of methanol using ultrasound. Subsequently, 1 mL of ammonia solution (25–28 wt%) was slowly added to the above dispersion under stirring. Then, 4 mL of AgNO_3_ solution (5 mg/mL) was added, followed by continuous stirring for 2 h to facilitate the reduction of Ag+ to Ag NPs. The resulting SNPB@Ag NPs were collected by centrifugation (12,000× *g*, 20 min), and then rinsed alternately with ethanol and distilled water. Finally, the SNPB@Ag NPs were freeze-dried or resuspended in distilled water for further use.

### 2.3. Formulation of GE/SNPB@Ag Hydrogel

Next, the thermo-sensitive hydrogel co-loaded with SNPB@Ag NPs and GE, along with its lyophilized powder, was prepared. Briefly, GE (0.2 g) was dissolved in 18 mL of PBS under stirring. Subsequently, 2 mL of SNPB@Ag solution (0.5 mg/mL) was added in the GE solution using ultrasound. Next, Poloxamer 407 (3.6 g) and carboxymethyl chitosan (0.1 g) was added to the above dispersion. Following stirring for 30 min under ice bath conditions, a thick, transparent blue hydrogel solution was obtained, referred to as GE/SNPB@Ag hydrogel, and subsequently stored below 8 °C. To form the lyophilized powder of the hydrogel, the obtained hydrogel was frozen at −20 °C for 12 h, and then lyophilized (−40 °C, below 10 Pa) for 12 h. The blue powder was stored in nitrogen-flushed pharmaceutical vials. In addition, both SNPB@Ag hydrogel and GE hydrogel were prepared using the same process. However, the SNPB@Ag hydrogel did not contain GE, while the GE hydrogel lacked SNPB@Ag.

### 2.4. Characterization of the Nanozyme Hydrogel

The porous structure of the freeze-dried GE/SNPB@Ag hydrogel, which was sprayed with gold layers, was analyzed using scanning electron microscopy (SEM) (Shimadzu, Kyoto, Japan). A particle analyzer (NanoBrook 90Plus, Brookhaven, NY, USA) was used to measure the hydrodynamic diameters and zeta potential of the prepared SNPB@Ag NPs. The morphology of the SNPB@Ag NPs was viewed by using a transmission electron microscopy (TEM) instrument (FEI, Long Island, NY, USA). The pH of the formulated GE/SNPB@Ag hydrogel was measured with an S3G pH meter (Leici, Shanghai, China). The viscosity and rheological performance of the GE/SNPB@Ag hydrogel were assessed using a rheometer (Bruker, Karlsruhe, Germany). The UV–Vis absorption spectra of the nanozyme were obtained using a UV-2600 spectrophotometer (Shimadzu, Japan). To investigate the in vitro release of GE from the composite hydrogel, 1 mL of the GE/SNPB@Ag hydrogel, containing 10 mg of GE, was encapsulated in dialysis bags (a molecular weight cutoff of 7000 Da). The release medium consisted of 10 mM PBS at a temperature of 37 ± 1.0 °C and shaken at 75 rpm. At selected time points, 5 mL of the release medium was taken. The filtered supernatant was analyzed using a spectrophotometer at 238 nm to determine the cumulative release of GE.

### 2.5. Photothermal Behavior of the Nanozyme

Firstly, the synthesized SNPB@Ag NPs were dispersed in PBS at different concentrations (0.025, 0.05, 0.1, and 0.2 mg/mL), and subsequently transferred to 2 mL Eppendorf tubes. Subsequently, the dispersions were subjected to irradiation with a laser (808-nm, 1 W/cm^2^), during which the temperature changes of the dispersions were measured. Next, the temperature variation of the SNPB@Ag NPs (0.05 mg/mL), irradiated under NIR laser at different power densities, was also measured. An S225 infrared thermal imaging camera (Fotric, Santa Clara, CA, USA) was applied to record the temperature variations. The photothermal stability of the SNPB@Ag NPs was obtained by repeating irradiation for 4 cycles. Finally, 1 mL of SNPB@Ag NPs was irradiated with laser to reach a stable temperature. Subsequently, the laser was turned off, allowing the dispersion to cool gradually to room temperature. The photothermal conversion efficiency (η) of the SNPB@Ag NPs was calculated using a previously reported method (see Supplementary Methods) [12,13].

### 2.6. NO and O_2_ Detection

Photothermal-responsive NO release was assessed using the Griess analysis kit [20,21]. Briefly, 4 mL of SNPB@Ag solution (0.2 mg/mL) was irradiated with a NIR laser (808-nm, 0.6 W/cm^2^). After laser irradiation, 0.4 mL of solution was taken and filtered through a microporous membrane. Then, 0.1 mL of Griess reagents I and II was mixed with 0.1 mL of the supernatant, followed by incubation at 37 °C for 10 min. The absorbance of the solution was determined at 540 nm using a UV–Vis spectrophotometer. The catalase-mimicking enzyme activity of SNPB@Ag NPs was investigated by measuring the amounts of dissolved O_2_ produced. Briefly, 10 mL of SNPB@Ag solution was mixed with 10 mL of PBS (pH = 7.4) containing H_2_O_2_ (50 mM). During the mixing process, the amount of dissolved O_2_ released was monitored using a JPB 607A dissolved oxygen detector (Leici, China).

### 2.7. Antibacterial Capability Assay

Methicillin-resistant *Staphylococcus aureus* (MRSA) and *Escherichia coli* (*E. coli*) were utilized to investigate the antibacterial capability of SNPB@Ag in vitro. The bacteria in the logarithmic growth phase were diluted to 2 × 10^6^ cfu/mL, and 0.1 mL of the diluted bacterial suspension was subsequently added to the wells of the 96-well plates. Next, an LB medium containing different concentrations of SNPB@Ag was blended with the bacteria, and the plates were incubated at 37 °C. The blank LB culture medium group served as the control group. In the irradiation group, laser irradiation (808 nm, 0.6 W/cm^2^) was applied for 5 min after 2 h of incubation. Subsequently, the bacterial suspension was diluted with sterile saline, and the mixture was evenly spread on LB agar plates. After incubation for 24 h, the number of viable bacterial colonies was assessed. The bacterial survival rate was calculated by dividing the colony-forming units (CFU) in the experimental groups by the CFU in the PBS control group.

### 2.8. In Vitro Antioxidant Activity

The ABTS assay was used to assess the total antioxidant capability of the GE/SNPB@Ag composite hydrogel. Following the combination of 2.45 mM potassium persulfate with 7 mM ABTS stock solution, the ABTS radical cation (ABTS+) was generated and subsequently diluted 40-fold with anhydrous ethanol. Subsequently, 2 mL of either the GE solution or the composite hydrogel was added to 2 mL of the ABTS·+ solution and incubated for 10 min. PBS was used in the control group. After filtering the membranes, the absorbance (optical density, *OD*) of the different sample groups at 734 nm was measured using a UV–Vis spectrophotometer. Sample preparation and testing were conducted in the dark. The radical scavenging capability is defined by the following equation: (*OD*_control_ – *OD*_sample_) × 100%/*OD*_control_.

### 2.9. Hemolysis Assay and Cytotoxicity Assay

Female Kunming mice aged 6–8 weeks were purchased from the Animal Center at Xuzhou Medical University for use in hemolysis experiments and the animal wound model. All experimental protocols on mice were conducted in accordance with the relevant regulations and guidelines of the Institutional Animal Care and Use Committee of Xuzhou Medical University. For the in vitro hemolysis analysis, blood samples were taken from mice and centrifuged at 3000× *g* for 10 min. The red blood cells were washed with PBS, and then resuspended in PBS. Subsequently, 2 mL of the 2% suspension was mixed with PBS, deionized water, or the composite hydrogel sample (dispersed in PBS) at varying doses. Deionized water and PBS were served as the positive control and negative control groups, respectively. After incubating at 37 °C for 4 h, each mixture was centrifuged, and the absorbance (*A*) of the supernatant was measured using a microplate reader at 540 nm. The hemolysis ratio is defined by the following equation: (*A*_sample_ − *A*_negative control_)/(*A*_positive control_ − *A*_negative control_).

The cytotoxicity of the composite hydrogel against HaCaT cells was evaluated using the CCK-8 assay. HaCaT cells were seeded in 96-well plates and incubated for 24 h. Subsequently, various concentrations of the composite hydrogel were added to the plates. After co-culturing at 37 °C for 24 h, aliquots of CCK-8 solution (10 μL) were introduced, and the plates were incubated at 37 °C for 2 h. The absorbance was then measured using a microplate reader at 450 nm. Cell viability was calculated as the ratio of the absorbance of the sample to that of the control. The control group was incubated with PBS.

### 2.10. In Vivo Wound Healing Experiments and Safety Evaluation

The infected wound mouse model was established according to previously reported procedures. To assess the in vivo photothermal capability of the nanozyme, mice were randomly divided into 5 groups (*n* = 3). Following inhalation of anesthesia with isoflurane (5%), the dorsal fur of the mice was partially shaved, and a circular and full-thickness skin wound (with a diameter of 1 cm) was created. Subsequently, 0.4 mL of MRSA suspension (1 × 10^8^ CFU/mL) was applied to the wound and smeared for 24 h to induce infection. After washing with saline, 0.1 mL each of PBS, SNPB@Ag solution, SNPB@Ag hydrogel, GE hydrogel, and GE/SNPB@Ag hydrogel were applied to the infected wounds. A laser (808 nm, 0.6 W/cm^2^) was then applied for 10 min. To assess the ability of the GE/SNPB@Ag hydrogel to promote wound healing in vivo, mice with MRSA infections in their back wounds were randomly assigned to 5 groups (*n* = 3). After washing with saline, 0.1 mL of PBS, GE hydrogel, SNPB@Ag solution, SNPB@Ag hydrogel (with NIR laser for 10 min), and GE/SNPB@Ag hydrogel (with NIR laser for 10 min) were applied to the infected wounds every two days. The body weights of the mice were recorded every two days. Moreover, tissue fluid was collected from the wound site using sterile cotton swabs on day 3, diluted with sterile saline, and then spread onto LB agar plates. After incubation, the number of viable bacterial colonies was assessed. Following the in vivo wound healing experiment, biochemistry assays and histopathological analyses were also conducted. Blood samples were obtained from both the PBS group and the composite hydrogel group. After centrifugation (4 °C), the supernatant was analyzed using a Chemray 240 automatic biochemical analyzer (Rayto, Shenzhen, China) to assess liver and renal function. Additionally, major organs from different groups were excised, fixed with 10% formalin, and then stained with H&E for histological analysis.

### 2.11. Statistical Analyses

The results are expressed as mean ± standard deviation (*n* ≥ 3). The data were statistically analyzed using GraphPad Prism software (9.0). A one-way analysis of variance (ANOVA) or Student’s t-test was conducted to identify statistical differences between groups. Statistical significance was defined as *p* < 0.05.

## 3. Results and Discussion

### 3.1. Development and Properties of Nanozyme and GE/SNPB@Ag Hydrogel

**Figure 1:**
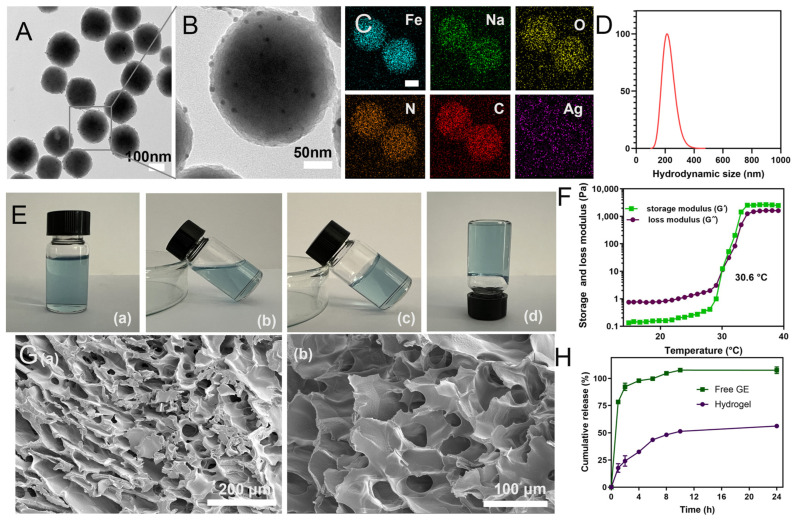
(**A**,**B**) TEM images of the nanozyme (**B** is a magnified version of **A**). (**C**) Elemental mapping of the nanozyme (scale bar, 50 nm). (**D**) Hydrodynamic diameter of the nanozyme. (**E**) Photographs of the composite hydrogel before (**a**,**b**) and after (**c**) phase transition, as well as storage for three months (**d**). (**F**) Rheological property of the composite hydrogel. (**G**) SEM images of the freeze-dried composite hydrogel (**a**,**b**). (**H**) In vitro drug release profiles of GE from the GE/SNPB@Ag hydrogel in PBS at pH 7.4. Each data point represents the mean ± SD (*n* = 3).

The thermo-sensitive GE/SNPB@Ag hydrogel was prepared using a two-step procedure. First, a multifunctional nanozyme, SNPB@Ag, was synthesized through a simple process. As shown in Figure 1A, the designed nanozyme exhibits a spherical shape and possesses a uniform size (about 0.2 μm). In addition, the magnified image in Figure 1B (derived from Figure 1A) clearly shows that very small Ag NPs (3–5 nm) are distributed on the surface of the SNPB@Ag nanozyme. Moreover, elemental analysis confirmed the composition of the nanozyme. Its internal structure is composed of iron, carbon, nitrogen, and oxygen, indicating that the core consists of SNPB, which is formed from sodium nitroprusside and potassium ferricyanide. Furthermore, the presence of silver on the exterior suggests that the nanozyme is decorated with Ag NPs (Figure 1C). Energy dispersive X-ray analysis of the SNPB@Ag nanozyme is presented in Figure S1. The proportion of Ag NPs in the nanozyme is approximately 4%. The hydrodynamic diameter and zeta potential of the nanozyme were 266 ± 10 nm and −35.9 ± 1.3 mV, respectively (Figure 1D and Figure S2). Meanwhile, the nanozyme exhibited a narrow polymer dispersity index of 0.067 ± 0.008. Additionally, the diameter of the nanozyme exhibited no significant change after being dispersed in PBS for four weeks, indicating that the nanozyme possesses good aqueous dispersity and stability. The ultraviolet (UV)–visible spectrum of the nanozyme is displayed in Figure S3.

In the second step, the synthesized SNPB@Ag nanozyme and the natural drug GE were embedded into a thermo-sensitive hydrogel to formulate a GE/SNPB@Ag composite hydrogel. As shown in Figure 1E, the composite hydrogel exhibits a blue appearance, which is attributed to the blue color of the nanozyme. The freeze-dried composite hydrogel possessed a three-dimensional porous sponge structure (Figure 1G). It is well known that the porous structure of hydrogels not only facilitates the absorption of exudates from ulcers and allows for oxygen permeation but also enhances the delivery of macromolecules, components of traditional Chinese medicine, cellular materials, and polymer NPs, among other substances [6,7,8]. Thus, the porous structure of the composite hydrogel can provide sufficient embedding space for the nanozyme and the natural drug GE. The composite hydrogel demonstrated low viscosity (less than 0.5 Pa·s) at room temperature, making it easy to spread on irregular wound surfaces [28,29]. After embedding the nanozyme and GE, the composite hydrogel retains the temperature-responsive characteristics of Poloxamer 407 hydrogel, exhibiting a rapid sol–gel phase transition (Figure 1E). The crosslinking in Poloxamer 407 hydrogels is primarily of a physical nature, arising from hydrophobic interactions, hydrogen bonds, and van der Waals forces. The increase in temperature causes the micelles of Poloxamer 407 to rearrange into a cubic structure, which subsequently transforms into a hexagonal structure. Thus, the formulated composite hydrogel is categorized as a type of physical gel [8,9]. Following this, rheological testing was performed. As depicted in Figure 1F, the storage modulus (G′) and the loss modulus (G″) represent the solid and fluid properties of the composite hydrogel, respectively [28]. When the temperature rose to 31 °C, the storage modulus (G′) of the hydrogel exceeded the loss modulus (G″), indicating that the composite hydrogel underwent a transition from a liquid state to a solid form. The gelation time for the composite hydrogel was 69 ± 2 s. In addition, the pH of the composite hydrogel was 6.4 ± 0.2. Given that the nanozyme can provide a significant photothermal effect even at a concentration of 0.05 mg/mL (as shown in the photothermal test), the concentrations of the nanozyme and GE in the composite hydrogel are established at 0.05 mg/mL and 10 mg/mL, respectively. Notably, as shown in Figure 1E(d), the nanozyme hydrogel retained its thermosensitive properties, and no significant changes were observed in its appearance or dispersion state after three months of storage. Given the sustained release properties of hydrogels, we subsequently investigated the release characteristics of GE from the composite hydrogel (Figure 1H). Due to the good water solubility of GE, the release of free GE is rapid in PBS (pH 7.4), reaching more than 90% within 2 h. However, the composite hydrogel demonstrated a sustained release manner, with less than 30% of GE released at 2 h and below 50% released at 10 h in PBS at pH 7.4. In addition, the composite hydrogel also displayed a controlled release manner in PBS at pH 5.0 (Figure S4). The controlled release characteristics of GE from the composite hydrogel would be beneficial for maintaining a high drug concentration at the wound site [30].

### 3.2. Photothermal Performance

**Figure 2:**
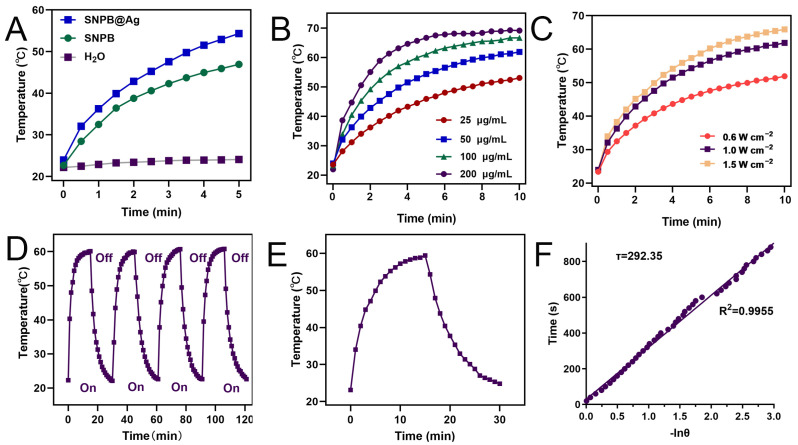
(**A**) Temperature changes curves of H_2_O, SNPB NPs, and SNPB@Ag nanozymes. (**B**) Temperature curves of the nanozyme at different concentrations. (**C**) Temperature curves of the nanozyme at different laser power densities (0.05 mg/mL). (**D**) Photothermal stability (0.05 mg/mL). (**E**) Temperature change of nanozyme (0.05 mg/mL, 1 W/cm⁻^2^) under NIR irradiation followed by a cooling period. (**F**) Plot of cooling time versus negative natural logarithm of the driving force acquired from (**E**).

PTT has already demonstrated advantages in antibacterial applications. Since both Ag- and Fe-based NPs exhibit photothermal properties, we investigated the photothermal characteristics of the synthesized SNPB@Ag nanozyme. Following 5 min of 808-nm laser illumination (1 W/cm⁻^2^, Figure 2A), the temperatures of the unmodified SNPB NPs and the nanozyme solution (1.0 mL, 0.05 mg/mL) were measured at 46 ± 1 °C and 54 ± 2 °C, respectively. In comparison to the unmodified SNPB NPs, the synthesized nanozyme exhibits an increased temperature change, attributed to the incorporation of Ag NPs. As shown in Figure 2B,C, the photothermal effect of the nanozyme is enhanced with increasing dosage (ranging from 0.025 mg/mL to 0.2 mg/mL) and laser power (ranging from 0.6 W/cm^2^ to 1.5 W/cm^2^). In addition, the nanozyme holds good photothermal stability after repeated laser illumination (Figure 2D). Figure 2E illustrates the temperature variations of the nanozyme dispersion (0.05 mg/mL) as it is heated to a steady-state concentration and subsequently cooled back to its initial temperature. Figure 2F displays the fitted cooling curve obtained from Figure 2E. The photothermal conversion efficiency (η) of the nanozyme was measured to be 50.6%. Benefiting from the stable photothermal properties of the nanozyme, the composite hydrogel-containing SNPB@Ag nanozyme can respond to an NIR laser to generate favorable photothermal effects for antibacterial applications.

### 3.3. NO-Generation and O_2_-Generation Performance

**Figure 3:**
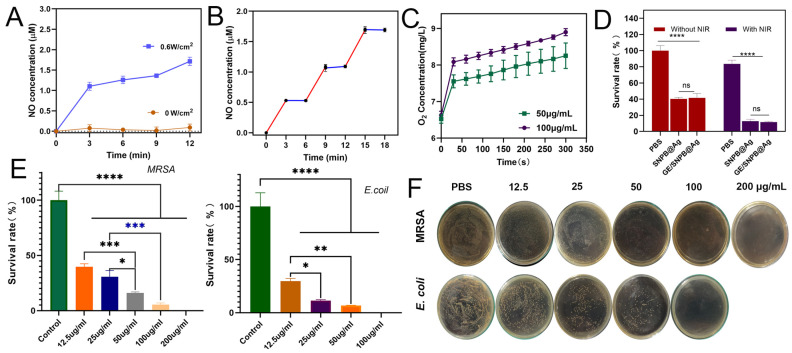
(**A**) Nitric oxide concentration changes after the irradiation of the nanozyme and (**B**) NIR laser irradiation: 0 to 3 min, 6 to 9 min, and 12 to 15 min. (**C**) Oxygen concentration changes in H_2_O_2_ solutions after adding the nanozyme at two concentrations. (**D**) Relative bacterial survival of MRSA treated with different samples. (**E**) Relative bacterial survival of MRSA and *E.coli* treated with the nanozyme under NIR laser irradiation (ns, *p* > 0.05; * *p* < 0.05; ** *p* < 0.01; *** *p* < 0.001; and **** *p* < 0.0001). (**F**) Representative photos of bacterial colonies formed by two typical bacteria.

NO is a vital gas molecule involved in various physiological and pathological processes. It is well known that NO is a potent vasodilator that enhances local blood circulation and increases blood flow. Additionally, NO can stimulate the migration and proliferation of endothelial cells, fibroblasts, and keratinocytes, thus facilitating the formation of microvessels. These processes ensure that the wound receives an ample supply of O_2_ and nutrients, which accelerates the healing process. Thus, the NIR laser-mediated NO generation performance of the synthesized nanozyme was assessed using the Griess analysis kit [20]. Under NIR laser irradiation, the synthesized nanozyme produces both a photothermal effect and releases NO (Figure 3A). During the 12 min of NIR laser irradiation, the production of NO increased progressively. Moreover, the release of NO ceases in the absence of an NIR laser (Figure 3B). This photothermal-responsive NO release resembles that observed in previously reported unmodified SNPB NPs [21]. An adequate O_2_ supply is also essential for promoting wound recovery. In the treatment of severe injuries or chronic wounds, O_2_ therapy (such as hyperbaric oxygen therapy) is employed to increase tissue O_2_ levels and facilitate healing [31]. Previous studies have reported that Prussian blue NPs exhibit catalase-mimicking enzyme activity, which allows them to decompose H_2_O_2_ into O_2_ and H_2_O [18]. Thus, the catalytic capacity of the nanozyme to produce O_2_ through the decomposition of H_2_O_2_ was also evaluated. Figure 3C illustrates that the dissolved O_2_ levels increased upon the addition of the nanozyme to an H_2_O_2_ aqueous solution (pH 7.4), suggesting that the nanozyme possesses favorable catalase-mimicking enzyme activity. Unlike the generation of NO, the production of O_2_ does not require stimulation from NIR laser. Leveraging its ability to generate both NO and O_2_, the nanozyme could consume excess H_2_O_2_, enhance blood flow, and facilitate the formation of microvessels at the wound site, thereby promoting wound healing [3,10].

### 3.4. Antibacterial Performance

The proliferation of microorganisms in wounds can inhibit tissue regeneration, resulting in delayed wound healing. Wound infections not only prolong healing time but may also lead to severe complications, such as sepsis. To investigate the antimicrobial activity of the synthesized nanozyme and its composite hydrogel, methicillin-resistant *Staphylococcus aureus* (MRSA) and *Escherichia coli* (*E. coli*) were selected as microbial models. For MRSA, the nanozyme exhibited antimicrobial capability even in the absence of an NIR laser, primarily due to the antibacterial activity of Ag NPs. Under NIR laser irradiation, the nanozyme demonstrated enhanced antimicrobial capability, which can be attributed to the synergistic effect between the photothermal properties of the nanozyme and the antibacterial activity of Ag NPs. Upon exposure to an NIR laser, the nanozyme exhibited an efficacy of over 90% in eliminating MRSA at a concentration of 0.1 mg/mL. Additionally, the composite hydrogel preserved the antibacterial capability of the nanozyme (Figure 3D). Next, the effect of nanozyme concentration on antibacterial activity was examined. The results suggest that the antibacterial efficiency of the nanozyme was concentration-dependent (Figure 3E). Encouragingly, the nanozyme coupled with NIR laser irradiation at a concentration of 0.1 mg/mL was able to eliminate all *E. coli.* Representative photographs of the spread plates are presented in Figure 3F. These results indicate that the developed nanozyme composite hydrogel holds potential as a novel antibacterial dressing for inhibiting wound bacteria [14].

### 3.5. In Vitro Antioxidant Activity

**Figure 4:**
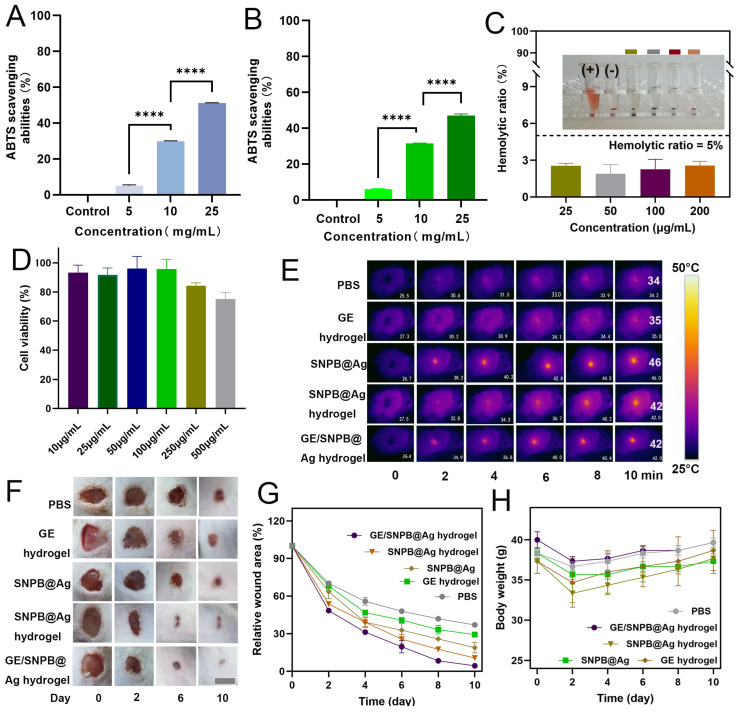
Radical scavenging activity of the GE solution (**A**) and the composite hydrogel (**B**) (**** *p* < 0.0001). (**C**) Hemolysis test of the composite hydrogel with different concentrations (*n* = 3). (**D**) Relative viability of HaCaT cells treated with the composite hydrogel (*n* = 3). (**E**) Thermal images of wound areas of mice treated with different samples. (**F**) Representative photos of wound areas of mice. Scale bar, 1 cm. (**G**) Changes in the wound areas of different mice groups. (**H**) Weight changes of different mice groups.

High levels of ROS can lead to oxidative stress, prolong the inflammatory response, and accelerate skin infections [25]. GE demonstrates anti-inflammatory and antioxidant properties, which help establish a conducive environment for early wound healing [26,27]. To evaluate the total antioxidant capacity of the composite hydrogel, the free radical scavenging efficiency of the 2,2′-azino-bis(3-ethylbenzthiazoline-6-sulfonic acid) radical cation (ABTS+) was investigated. As shown in Figure 4A, the GE solution demonstrates concentration-dependent radical scavenging activity (ranging from 5 mg/mL to 25 mg/mL). The ABTS·+ scavenging rate reaches approximately 30% at a concentration of 10 mg/mL. As expected, the composite hydrogel also possesses similar radical scavenging activity (Figure 4B). Therefore, the GE-loaded composite hydrogel, which exhibits favorable antioxidant activity, has significant potential as a ROS scavenger for biomedical applications.

### 3.6. Hemostasis and Cytocompatibility

To assess its potential for biomedical applications, we evaluated the hemocompatibility and cytotoxicity of the composite hydrogel. The hemolysis ratios of the hydrogel are quantitatively presented in Figure 4C. Within the concentration range of 25 to 200 μg/mL, the composite hydrogel did not compromise the integrity of the erythrocyte membrane. Even at the highest concentration tested (200 μg/mL), the hemolysis ratio remained below 5%. Following this, the cytotoxicity of the composite hydrogel against human skin keratinocytes (HaCaT) cells was assessed using the CCK-8 assay. As revealed in Figure 4D, cell viability remained unaffected when the composite hydrogel concentration increased to 0.5 mg/mL, indicating that the composite hydrogel exhibits low cytotoxicity. The results of cytotoxicity and hemolysis assays indicate that the composite hydrogel has high biocompatibility.

### 3.7. In Vivo Therapeutic Effect for Wound Healing

To demonstrate the in vivo photothermal capability of the nanozyme, we assessed its photothermal performance on infected wounds. Following the application of 0.1 mL each of PBS, SNPB@Ag solution, the SNPB@Ag hydrogel, the GE hydrogel, and the GE/SNPB@Ag hydrogel to the infected wounds, a laser (808 nm, 0.6 W/cm^2^) was applied for 10 min. As depicted in Figure 4E and Figure S5, the temperature changes in the wound areas of both the PBS group and the GE hydrogel were minimal during NIR laser irradiation, owing to the absence of active photothermal components. In contrast, the wound areas of mice treated with the SNPB@Ag solution, the SNPB@Ag hydrogel, and the GE/SNPB@Ag hydrogel exhibited a significant increase in temperature following 10 min of irradiation, attributable to the photothermal properties of SNPB@Ag. Due to the dispersion effect of the hydrogel layer, the SNPB@Ag hydrogel and the GE/SNPB@Ag hydrogel demonstrated a more moderate photothermal response compared to the SNPB@Ag solution, thereby reducing the risk of thermal injury to the wound. The photothermal properties of the hydrogel affords several advantages for promoting wound healing. First, the resultant photothermal effect enhances local blood circulation. Additionally, this photothermal effect can synergistically enhance the antibacterial action of Ag NPs, aiding in bacterial eradication. Furthermore, it exhibits a significant role in stimulating the release of NO, which promotes wound healing.

Next, the ability of the GE/SNPB@Ag hydrogel to promote wound healing in vivo was investigated. Mice with MRSA infections in their back wounds were randomly assigned to five groups (*n* = 3). After washing with saline, 0.1 mL of PBS, GE hydrogel, SNPB@Ag solution, SNPB@Ag hydrogel, and GE/SNPB@Ag hydrogel were separately applied to the infected wounds and irradiated with or without an NIR laser every two days. Figure 4F presents representative images depicting the changes in infected wounds across different groups over ten days. Following treatment with various formulations, all groups exhibited a reduction in wound size, which is associated with their self-repair capabilities. However, the wounds in the control group, the GE hydrogel group, and the SNPB@Ag solution without an NIR laser remained distinctly visible. Notably, the GE/SNPB@Ag hydrogel and laser irradiation group demonstrated a significantly accelerated healing process, nearing complete recovery by day 10 (Figure 4G). Moreover, the bacterial burden at the wound site after the second treatment was assessed by quantifying the number of bacterial colonies on Luria-Bertani (LB) agar plates. As illustrated in Figure S6, the PBS group displayed a high bacterial load at the wound site due to the lack of antimicrobial components. In contrast, bacterial colonies in both the SNPB@Ag hydrogel combined with the laser irradiation group and the GE/SNPB@Ag hydrogel combined with the NIR laser irradiation group were dramatically reduced. These results are consistent with the results of the in vitro antibacterial experiments. The significant therapeutic effect in the GE/SNPB@Ag hydrogel group can largely be attributed to the photothermal effect, NO generation, O_2_ generation, and the efficient bacteria-killing ability conferred by the embedded nanozyme. In addition, the hydrogel provides a moist and breathable environment for the wound, and possesses controlled drug release capabilities, which enhances its therapeutic effect [5,6]. In the absence of laser irradiation, however, the SNPB@Ag solution does not exhibit photothermal performance, nor its associated antibacterial properties and NO supply characteristics [20]. On the other hand, the healing effectiveness of the GE/SNPB@Ag hydrogel group is superior to that of the SNPB@Ag hydrogel group, which can be attributed to the effects of GE. It is widely recognized that ROS plays a vital role in tissue injury, repair, and regeneration. GE has antioxidant and anti-inflammatory properties, which help establish a conducive environment for wound healing [32,33]. Additionally, GE can promote collagen synthesis, thereby enhancing the mechanical strength and integrity of the wound [25,26]. Therefore, these results demonstrate that the composite hydrogel combined with laser irradiation is an effective method for accelerating the healing of infected wounds.

### 3.8. In Vivo Safety Evaluation

**Figure 5:**
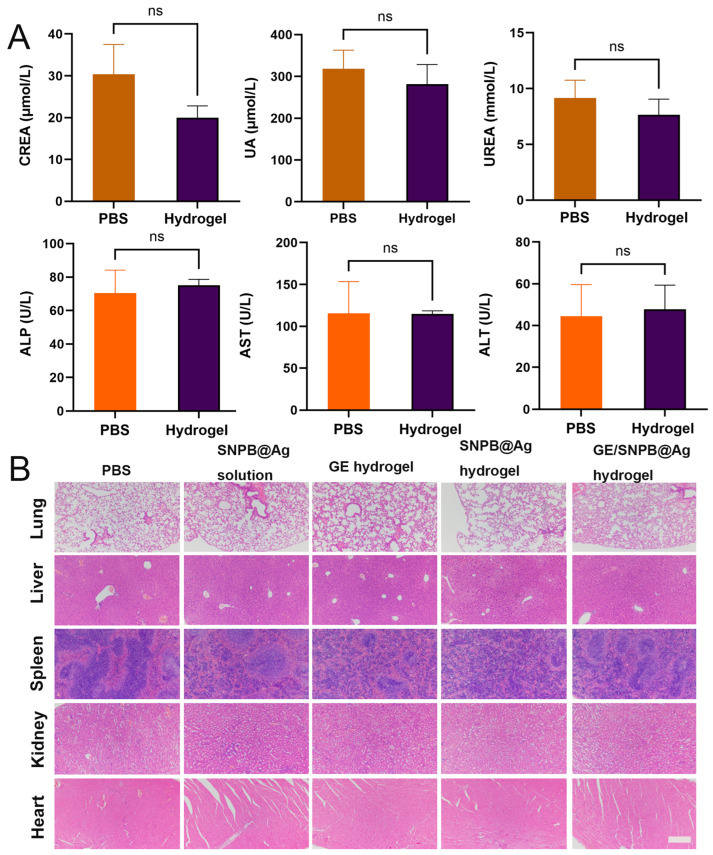
(**A**) Liver and kidney function of the PBS-treated mice and the composite hydrogel-treated mice (*n* = 3) (ns, *p* > 0.05). (**B**) H&E staining of major organs of different mice groups. Scale bar, 100 μm.

Apart from the ability to promote wound healing and possession of antibacterial properties, a wound dressing must also be appropriately safe. During the treatment, no significant fluctuations in body weight were observed in any of the groups (Figure 4H). To further assess the safety of the developed composite hydrogel, blood biochemical and histological analyses were also conducted. After the wound healing study, blood samples were taken from both the PBS group and the composite hydrogel group. Next, a series of markers were measured to investigate hepatotoxicity and nephrotoxicity of the composite hydrogel. As shown in Figure 5A, no obvious differences in these markers were observed between the composite hydrogel group and the control group. This suggests that the composite hydrogel did not cause significant acute kidney or liver toxicity. Furthermore, we conducted histological analysis. Hematoxylin-eosin (H&E) staining demonstrated that the major tissues from the mice in the composite hydrogel group maintained normal physiological morphology, with no apparent pathological damage or inflammatory response (Figure 5B). These results indicate that the composite hydrogel exhibits favorable biosafety, making it a promising and effective platform for wound dressing and promoting infected wound healing.

## 4. Conclusions

In summary, a novel type of versatile nanozyme (SNPB@Ag), along with GE, was encapsulated in a thermo-sensitive hydrogel composed of Poloxamer 407 and carboxymethyl chitosan. This composite hydrogel integrates photothermal properties, photothermal-induced NO release, antibacterial activity, catalase activity, and antioxidative properties into a single platform. Additionally, the composite hydrogel demonstrates temperature responsiveness and sustained drug release capabilities. Due to these advantages, the composite hydrogel exhibited significant therapeutic effects in a mouse model of acute wound infections. Furthermore, the composite hydrogel displays high biocompatibility. These results suggest that the GE/SNPB@Ag hydrogel provides a novel approach for the treatment of infected wounds.

## Data Availability

The original contributions presented in this study are included in the article/Supplementary Materials. Further inquiries can be directed to the corresponding author.

## Supplementary Materials

The following supporting information can be downloaded at: https://www.mdpi.com/article/10.3390/pharmaceutics16121624/s1, Figure S1: Energy dispersive X-ray analysis of the nanozyme; Figure S2: The zeta potential of different samples (*n* = 3); Figure S3: The UV–visible spectrum of (a) SNPB NPs and (b) SNPB@Ag NPs; Figure S4: In vitro drug release profiles of GE from the GE/SNPB@Ag hydrogel in PBS at pH 5.0; Figure S5: Temperature changes at the wound areas of mice (*n* = 3); Figure S6: Relative bacterial survival of MRSA treated with PBS, GE hydrogel, SNPB@Ag solution, SNPB@Ag hydrogel (with NIR laser), and GE/SNPB@Ag hydrogel (with NIR laser) (*n* = 3).
