# Supplementary material for: A Multifunctional Nanozyme Hydrogel with Antibacterial, Antioxidative, and Photo-Induced Nitric Oxide-Supplying Properties for Promoting Infected Wound Healing
Source: Pharmaceutics. 2024 Dec 22;16(12):1624. doi: 10.3390/pharmaceutics16121624 (PMC11678166; doi:10.3390/pharmaceutics16121624)
Supplement: Supplementary file 1 [file pharmaceutics-16-01624-s001.zip › pharmaceutics-3349959-supplementary.pdf]

A Multifunctional Nanozyme Hydrogel with Antibacterial, Antioxidative, and Photo-Induced Nitric Oxide-Supplying Properties for Promoting Infected Wound Healing

Equations for photothermal conversion efficiency (η)

Obtained time constant (τs) for heat transfer of the photothermal agents by applying linear time data versus ln θ from the cooling stage. θ can be calculated according to the equation (S1):

$$\theta = \frac{T - T_{surr}}{T_{max} - T_{surr}} \qquad \text{Equation (S1)}$$

The photothermal conversion efficiency (η) of the NPs can be calculated according to the equation (S2):

$$\eta = \frac{hS(T_{max} - T_{surr}) - Q_{dis}}{I(1 - 10^{-A_{808}})} \times 100\% \qquad \text{Equation (S2)}$$

The maximum steady temperature (T_{Max}) and environmental temperature (T_{Surr}) were 59.4 °C and 23.1 °C, respectively. h is the heat transfer coefficient, S is the surface area of the container, and the value of hS can be calculated by MC/τs. Where M is the mass of water (1 g), C is the specific heat capacity of water (4.2 J/g °C), and τs is the time constant (292.35 s). The Q_{dis} represents the heat dissipated from the light absorbed by the container, and it is calculated to be approximately equal to 0.02 W. The incident laser power (I) and the absorbance of the NPs dispersion at 808 nm (A808) were 1 W and 1.925, respectively.

Formula for cumulative release

$$W\% = \frac{C_{ti} \times V + \sum C_{ti} \times V_{ti}}{M} \times 100\%$$

W%: Cumulative release amount of the drug, *M*: Initial drug loading, *Cti*: Concentration of the drug in the extracted sample at time *ti*, *V*: Volume of *release* medium, *Vti*: Volume of extracted sample.

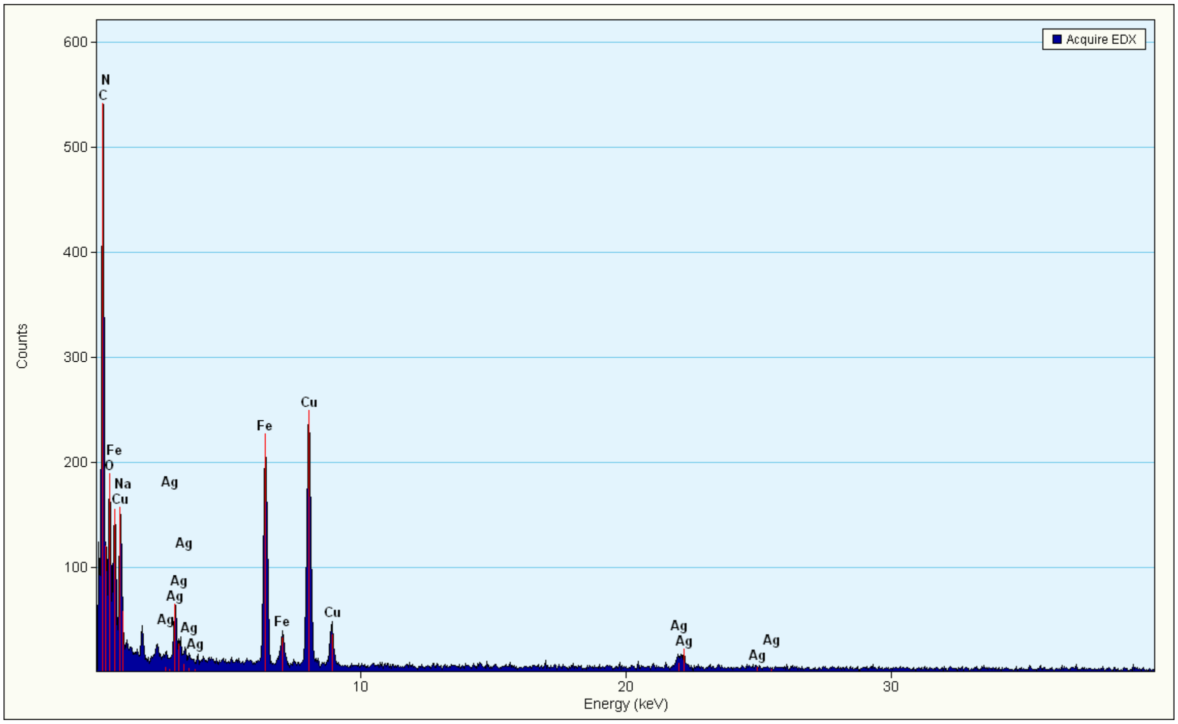

Figure S1. Energy dispersive X-ray analysis of the nanozyme.

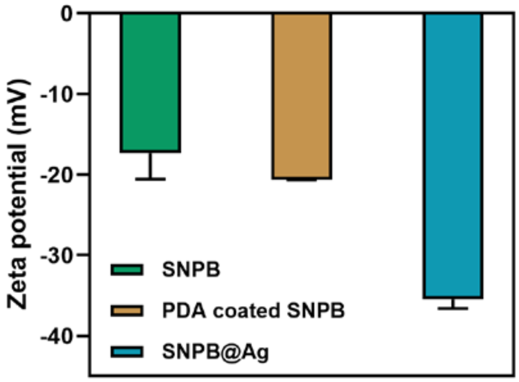

Figure S2. The zeta potential of different samples (n=3).

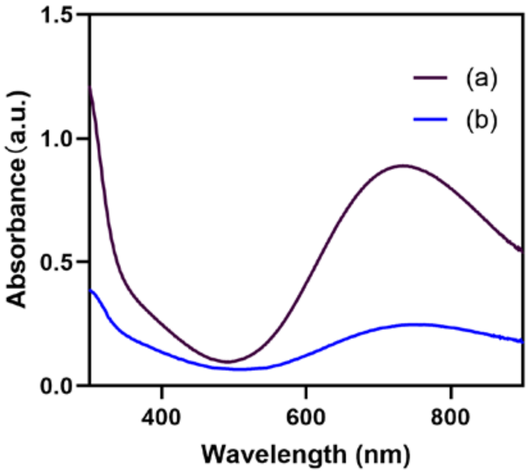

Figure S3. The UV-visible spectrum of (a) SNPB NPs and (b) SNPB@Ag NPs.

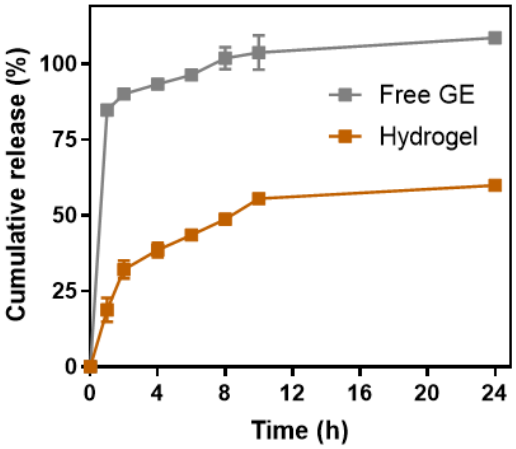

Figure S4. *In vitro* drug release profiles of GE from the GE/SNPB@Ag hydrogel in PBS at pH 5.0 (n = 3).

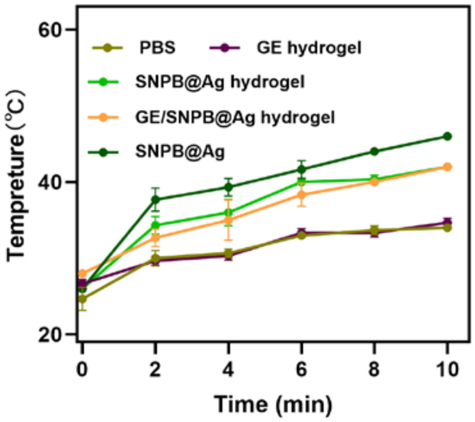

Figure S5. Temperature changes at the wound areas of mice (n = 3).

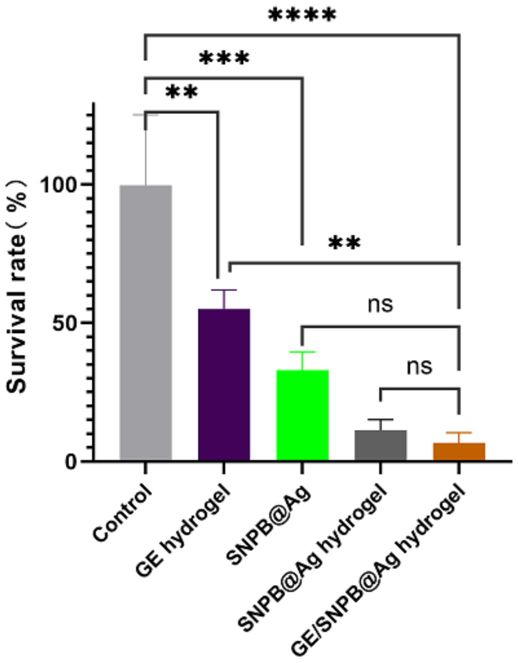

Figure S6. Relative bacterial survival of MRSA treated with PBS, GE hydrogel, SNPB@Ag solution, SNPB@Ag hydrogel (with NIR laser), and GE/SNPB@Ag hydrogel (with NIR laser) (n = 3).
